# Differential Expression Analysis of a Subset of Drought-Responsive *GmNAC* Genes in Two Soybean Cultivars Differing in Drought Tolerance

**DOI:** 10.3390/ijms141223828

**Published:** 2013-12-06

**Authors:** Nguyen Phuong Thao, Nguyen Binh Anh Thu, Xuan Lan Thi Hoang, Chien Van Ha, Lam-Son Phan Tran

**Affiliations:** 1 School of Biotechnology, International University, Vietnam National University HCMC, Quarter 6, Linh Trung Ward, Thu Duc District, Ho Chi Minh 70000, Vietnam; E-Mails: thunba05@mp.hcmiu.edu.vn (N.B.A.T.); htlxuan@hcmiu.edu.vn (X.L.T.H.); 2 Signaling Pathway Research Unit, RIKEN Center for Sustainable Resource Science, 1-7-22, Suehiro-cho, Tsurumi, Yokohama 230-0045, Japan; E-Mail: chienhavan@psc.riken.jp; 3 National Key Laboratory of Plant Cell Biotechnology, Agricultural Genetics Institute, Vietnamese Academy of Agricultural Science, Pham Van Dong Street, Hanoi 10000, Vietnam; 4 Post-Graduate Program, Vietnamese Academy of Agricultural Science, Thanhtri, Hanoi 10000, Vietnam

**Keywords:** comparative expression analysis, drought stress, NAC transcription factor, real-time quantitative PCR, soybean

## Abstract

The plant-specific NAC transcription factors play important roles in plant response to drought stress. Here, we have compared the expression levels of a subset of *GmNAC* genes in drought-tolerant DT51 and drought-sensitive MTD720 under both normal and drought stress conditions aimed at identifying correlation between *GmNAC* expression levels and drought tolerance degree, as well as potential *GmNAC* candidates for genetic engineering. The expression of 23 selected dehydration-responsive *GmNAC*s was assessed in both stressed and unstressed root tissues of DT51 and MTD720 using real-time quantitative PCR. The results indicated that expression of *GmNAC*s was genotype-dependent. Seven and 13 of 23 tested *GmNAC*s showed higher expression levels in roots of DT51 in comparison with MTD720 under normal and drought stress conditions, respectively, whereas none of them displayed lower transcript levels under any conditions. This finding suggests that the higher drought tolerance of DT51 might be positively correlated with the higher induction of the *GmNAC* genes during water deficit. The drought-inducible *GmNAC011* needs to be mentioned as its transcript accumulation was more than 76-fold higher in drought-stressed DT51 roots relative to MTD720 roots. Additionally, among the *GmNAC* genes examined, *GmNAC085*, *092*, *095*, *101* and *109* were not only drought-inducible but also more highly up-regulated in DT51 roots than in that of MTD720 under both treatment conditions. These data together suggest that *GmNAC011*, *085*, *092*, *095*, *101* and *109* might be promising candidates for improvement of drought tolerance in soybean by biotechnological approaches.

## 1. Introduction

Drought is one of the most devastating abiotic stresses, which negatively impacts plant growth and development [1]. In response to water deficit, plants trigger a number of physiological and metabolic processes to promote their survival [2,3]. At a molecular level, upon perceiving the environmental stress signal, numerous genes in plants, including those encoding transcription factors (TFs), have altered their expression for stress adaptation [4]. TFs have been known to play important roles in plant stress responses by regulating various signaling pathways through their binding to the *cis*-acting element(s) located in promoter region of downstream target genes, thereby activating them, and/or through interaction with other proteins [5]. A significant number of TFs, such as those belonging to AP2/ERF (Apetala 2/ethylene-responsive element binding factor), bZIP (basic-domain leucine zipper), MYB (myeloblastosis), WRKY, and NAC (NAM—no apical meristem, ATAF—*Arabidopsis* transcription activation factor, and CUC—cup-shaped cotyledon) families, have been reported to be involved in regulation of drought stress responses [6–10].

The plant-specific NAC TF family was first described in *Petunia* more than 15 years ago [11]. In the last decade, advances in genomic sequencing have allowed the research community to identify the *NAC* family members in a number of sequenced species, such as 117 genes in *Arabidopsis*, 151 in rice (*Oryza sativa*) [12], 163 in poplar (*Populus trichocarpa*) [13], 152 in tobacco (*Nicotiana tabacum*) [14], and approximately 200 members in soybean (*Glycine max*) [15]. The NAC TFs are multi-functional proteins and involved in diverse processes, including auxin signaling and lateral root formation [16], embryo development [17], flowering [18], regulation of secondary cell wall synthesis, cell division [19], biotic and abiotic stress responses [20,21]. In general, the NAC TFs share a conserved DNA-binding domain located at the *N*-terminal end, and a variable domain at the *C*-terminal end important for the transcriptional regulatory functions [17,22–24]. In drought stress signaling, NAC TFs are involved in both abscisic acid (ABA)-dependent and ABA-independent pathways [2]. The involvement of NAC TFs in regulation of drought response was first reported in *Arabidopsis* with the discovery of the multiple stress-responsive *ANAC019*, *ANAC055* and *ANAC072* genes, whose overexpression significantly improved drought tolerance of *Arabidopsis* transgenic plants [10]. Following this study, a number of *NAC* genes have been identified in various species, including crop plants, such as *OsNAC6*, *SNAC1* and *ONAC45* in rice [21,25–27], *TaNAC69* and *TaNAC2a* in wheat (*Triticum aestivum*) [28,29], and *AhNAC3* in peanut (*Arachis hypogaea*) [30], which showed strong potential for genetic engineering of improved stress-tolerant crops.

Soybean is a nutritionally important crop due to its great supplies of protein- and oil-rich food for human consumption and animal feed [31]. According to the Food and Agricultural Organization of United Nations’s statistic (2012), worldwide soybean production is more than 250 million metric tons, mainly from United States, Brazil, Argentina, China and India. Vietnam, with distribution of 175,295 metric tons, also belongs to the top 25 soybean-producing countries [32]. However, drought stress has led to significant reductions in soybean yield (24%–50%) at various locations over the world [33,34]. To cope with drought stress, in recent years intensive research has been conducted to gain a better insight into molecular mechanisms underlying drought responses in soybean, especially at transcriptional and translational levels to discover and functionally analyze the genes involved [1,35–38]. Thanks to the completion of the soybean genomic sequence in 2010, at least 61 TF families were identified in soybean by computational prediction, among which the NAC TF family was predicted to consist of more than 180 members by several research groups [15,39,40]. The first six *GmNAC* genes called *GmNAC1–6* were identified by Meng *et al*. in 2007 [41], and the expression of these genes under osmotic stress was thoroughly examined [42]. Later on, in the first large-scale study of the *GmNAC* family, expression profiling of 31 *GmNAC* genes in soybean seedlings demonstrated that 9 *GmNAC* genes were induced by dehydration, high salinity, cold and/or ABA treatments [31]. More recently, a comprehensive analysis of *GmNAC* family by Le *et al*. (2011) identified 152 full-length GmNAC TFs, including 11 membrane-bound members within soybean genome [43]. Furthermore, out of 38 *GmNAC* genes the authors found 25 and 6 *GmNAC*s induced and repressed 2-fold or more, respectively, in roots and/or shoots of soybean seedlings by dehydration treatment using real-time quantitative PCR (RT-qPCR). In addition, the same group demonstrated the complexity in the dynamics of drought-responsive expression of the *GmNAC* genes as they reported that several *GmNAC* genes displayed different drought-responsive expression profiles in different tissues at the same development stage or in the same tissue at different development stages [44]. Strong lines of evidence obtained from various model and crop plants, including soybean, collectively suggest that NAC TFs play an important role in plant adaptation to various stresses, thereby providing novel tools and resources for improvement of stress tolerance in economically important crops.

In spite of their important roles in plant responses to stresses, the potential of GmNACs has not been fully explored in soybean. In this study, we have carried out differential expression analysis of 23 selected dehydration-responsive *GmNAC* genes in DT51 (a drought-tolerant soybean variety) and MTD720 (a drought-sensitive soybean variety) that have contrasting drought-responsive phenotypes. We hypothesized that differential expression of *GmNAC* genes might contribute to drought tolerance of DT51 *versus* MTD720. Therefore, we examined the correlation between expression of *GmNAC* genes and drought tolerance degrees to identify possible role of *GmNACs* in DT51 that has enhanced drought-tolerant trait. Additionally, the differentially expressed *GmNAC*s could serve as potential genetic resources for development of soybean elite cultivars with improved drought tolerance by genetic engineering.

## 2. Results and Discussion

### 2.1. Differential Expression of a Subset of Drought-Responsive *GmNAC* Genes in the Roots of DT51 and MTD720

Previously, among 152 *GmNAC*s, which have putative full-length open reading frame, 50 stress-related genes were predicted based on phylogenetic analyses of *GmNAC*, *ANAC* and *ONAC* families [43]. Out of these 50 genes, 38 genes were checked by RT-qPCR, and 25 up-regulated and 6 down-regulated genes were identified in roots and/or shoots of 12-day-old soybean seedlings in response to dehydration [43]. To determine whether the differential expression of *GmNAC* genes would contribute to enhanced drought tolerance we have carried out differential expression analysis of selected genes in a drought-tolerant (DT51) and in a drought-sensitive soybean variety (MTD720). These varieties have contrasting drought-tolerant phenotype under the same experimental conditions by comparing their relative water content (RWC) and drought-tolerant index (DTI) with 11 other soybean cultivars (data submitted for publication to BioMed Research International, Thu *et al.*). In addition, under non-stressed conditions, DT51 has longer root and shoot lengths than MTD720, especially at V3-stage (not shown). From the identified 31 dehydration-responsive *GmNAC* genes [43], we selected 23 genes that displayed the highest expression change by dehydration treatment, including 17 up-regulated and all 6 down-regulated genes, grouped to Group A and B in this work, respectively, for comparative expression analyses (Table S1).

Root plasticity has been considered as an important physiological trait in genotypic adaptation to drought stress. Plants with desirable root traits, such as longer primary root and/or larger lateral root system, can adapt better to drought stress as they can reach water at lower soil layers and forage subsoil surface moisture [45–47]. Since regulation of root plasticity has been implicated as one of the most important activities reflecting the plant responses against drought stress, in this study we had the highest interest in analyzing differential expression of the selected *GmNAC* genes in roots of DT51 and MTD720 to identify the possible correlation between enhanced drought tolerance of DT51, as well as potential *GmNAC* genes for genetic engineering.

**Figure 1.**
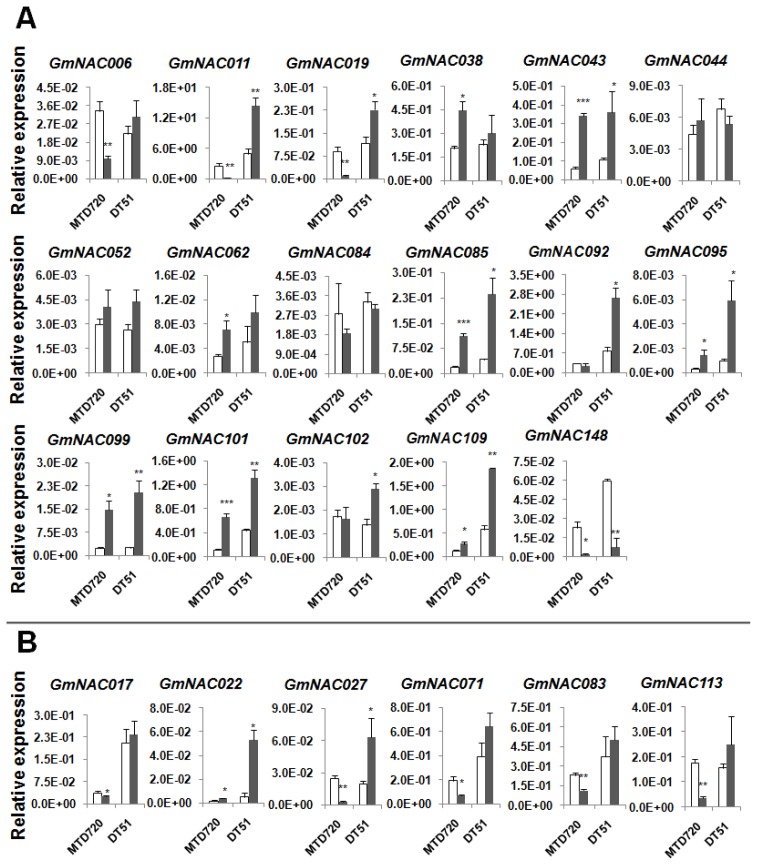
Expression of 17 selected dehydration-inducible (**A**) and six selected dehydration-repressible (**B**) *GmNAC* genes in roots of MTD720 and DT51 under normal (white bars) and drought (black bars) conditions. Asterisks on the top of bars indicate statistically significant differences (*p-value < 0.05; **p < 0.01; ***p < 0.001) between treated and untreated root samples within a cultivar.

Using the criteria of the ratio change ≥2 and *p*-value < 0.05, out of 23 selected dehydration-responsive *GmNAC*s a total of 19 genes displayed altered expression in roots of DT51 and/or MTD720 after the drought treatment (Figure 1, Table 1). We found that among 14 genes of Group A, 9 genes (*GmNAC011*, *043*, *085*, *092*, *095*, *099*, *101*, *102* and *109*) were up-regulated in DT51 roots, whereas *GmNAC148* was down-regulated as a consequence of the drought treatment (Figure 1A, Table 1). The remaining 4 genes of Group A (*GmNAC006*, *019*, *038* and *062*) did not show significant transcriptional change under our experimental conditions. As for the *GmNAC* genes in Group B, our data showed that *GmNAC022* and *027* were induced, while the expression of the remaining genes (*GmNAC017*, *071*, *083* and *113*) was considered as unaltered in drought-treated roots of DT51 based on the predefined criteria (Figure 1B, Table 1).

In drought-sensitive MTD720, as shown in Figure 1A and Table 1, 12 genes in Group A displayed significant expression change in the roots under drought stress. Among these genes, apart from *GmNAC006*, *011*, *019* and *148*, whose expression was assigned as down-regulated, 8 remaining genes, including *GmNAC038*, *043*, *062*, *085*, *095*, *099*, *101* and *109*, showed up-regulation in response to drought stress. The remaining two genes of Group A (*GmNAC092* and *102*) did not have their gene expression changed during drought stress. Regarding to the *GmNAC*s in Group B, 4 genes (*GmNAC027*, *071*, *083* and *113*) were down-regulated, while *GmNAC022* was induced in drought-treated MTD720 roots (Figure 1B, Table 1).

The diverse dehydration/drought-responsive expression of *GmNAC* genes observed in the roots of DT51 and MTD720 might suggest that the expression of *GmNAC* genes is genotype-dependent. This diversification would suggest that there might be a positive correlation between drought-responsive expression levels of *GmNAC* genes and drought tolerance degrees of DT51 and MTD720, as more up-regulated and less down-regulated genes were identified in drought-treated roots of DT51, whereas the opposite tendency was observed in that of MTD720 (Table 1). Specifically, among 19 *GmNAC* genes with altered drought-responsive expression in DT51 and/or MTD720, there were 11 up-regulated, 7 unaltered and only 1 down-regulated genes in drought-stressed roots of DT51, whereas 9 up-regulated, 2 unaltered and 8 down-regulated genes were found in the respective tissues of MTD720.

As a means to find further evidence for the existence of the positive correlation between the expression levels of the examined *GmNAC* genes and the different tolerance degrees of DT51 and MTD720, we compared the expression levels of the tested *GmNAC*s in the root tissues of the two cultivars under both normal and drought conditions. Our data showed that under unstressed conditions, 7 genes, namely *GmNAC085*, *092*, *095*, *101*, *109* and *148* of Group A, and *GmNAC017* of Group B, had significantly higher expression levels in DT51 roots relative to MTD720 roots (Table 2). The expression levels of the remaining genes did not significantly differ in DT51’s and MTD720’s untreated root tissues. On the other hand, there were more genes, 13 out of 23 tested *GmNAC*s, showing significantly higher expression levels in roots of DT51 than in that of MTD720 under drought stress. These included *GmNAC006*, *011*, *019*, *085*, *092*, *095*, *101* and *109* of Group A, and *GmNAC017*, *022*, *027*, *071* and *083* of Group B. Not a single *GmNAC* gene was observed to have higher expression in MTD720 roots relative to DT51 roots under either condition. In addition, we found that *GmNAC085*, *095*, *101* and *109* were not only drought-inducible in roots of DT51 and MTD720 but also had higher transcript levels in roots of DT51 than in that of MTD720 under both normal and drought conditions (Tables 1 and 2). It is also worthy to note that *GmNAC011* and *027* exhibited opposite tendency in regulation between the two varieties. Since these two genes were induced in drought-stressed roots of DT51 but repressed in the respective tissues of MTD720, *GmNAC011* and *027* showed more than 76- and 20-fold higher expression, respectively, in roots of DT51 than in that of MTD720 under drought stress (Figure 1; Table 1). Taken together, these results suggest that the better drought tolerance of DT51 is, at least in part, associated with the enhanced expression of a subset of the *GmNAC* genes under normal and/or drought stress conditions.

### 2.2. Potential Drought-Responsive Genes for Genetic Engineering

One of the objectives of this study was to identify drought-responsive genes that could be used for development of soybean cultivars with improved drought tolerance via genetic engineering technologies. One strategy, which is often used to identify promising candidate genes, is differential expression analysis of cultivars with contrasting drought-tolerant phenotypes [35]. Thus, taking the advantage of this study we searched for *GmNAC* genes that have more highly expression levels in DT51 than in MTD720, especially under stress conditions [35]. Furthermore, on the basis of the hypothesis that a potential candidate gene should be applicable to any genotype to overcome the drought stress and the genetic engineering approach could be either over-expression of knockdown, the candidate gene should be in general either induced or repressed in response to drought in both contrasting cultivars [35]. The candidate genes may also be those that are unaltered in drought-sensitive cultivar but induced in drought-tolerant cultivar, or repressed in drought-sensitive genotype and unaltered or induced in drought-tolerant genotype [48].

According to these principles, we have identified a number of genes that can be considered for further functional characterizations prior to using them in genetic engineering. When comparing the gene expression levels in the root tissues of the two cultivars exposed to drought stress, *GmNAC085*, *095*, *101* and *109* were drought-inducible in root tissues of both cultivars and showed significantly higher expression levels in DT51 not only under drought but also under normal conditions (Tables 1 and 2). Among these 4 genes, of particular interest is *GmNAC085* since it shares 39% identity at protein level with *SNAC1*, a rice *NAC* gene, whose overexpression enhanced drought tolerance of transgenic rice plants under field conditions [26]. *GmNAC011* (*GmNAC20* in [22]) is also a very attractive candidate gene as its overexpression in *Arabidopsis* was shown to enhance salt tolerance and improved lateral root development [22], that could thus benefit plant adaptation to drought stress as well [45–47]. The expression level of *GmNAC011*, which was found to be up- and down-regulated in drought-stressed roots of DT51 and MTD720, respectively, was approximately 76.6-fold higher in DT51 roots than in that of MTD720 under drought stress. *GmNAC092* (previous named *GmNAC4* in [31,41,42]), which was up-regulated in drought-stressed roots of DT51 but unaltered in that of MTD720 and expressed at higher level in DT51 roots under both conditions, can be considered a potential candidate gene, too. *GmNAC092* was grouped together with *ANAC019*, *ANAC055* and *ANAC072* in phylogenetic analyses [41,43], which were demonstrated to enhance drought tolerance when overexpressed in transgenic *Arabidopsis* plants [10].

In summary, we identified six potential candidate genes including *GmNAC011*, *085*, *092*, *095*, *101* and *109*. Not only their coding regions, but also their drought-inducible promoters may be promising for genetic engineering. Increasing evidence has indicated that application of stress-inducible promoters in biotechnology can overcome the negative effect of excessive overproduction of the protein resulted from the usage of constitutive promoters [49,50]. To gain an insight into the potential applications of the promoters of these candidate *GmNAC* genes in transgenic technologies under various stress conditions, we have searched for the well-known stress-responsive *cis*-motifs, including ABRE1 (ABA responsive element 1), ABRE2 (ABA responsive element 2), CE1 (coupling element 1), CRT (C-repeat), ICEr1 (induction of CBF expression region 1), ICEr2 (induction of CBF expression region 2), LTRE (low temperature-responsive elements), MYBR (MYB recognition site), MYCR (MYC recognition site), NACR (NAC recognition site) [8,51], G-box [52], CE3 (coupling element 3), DRE (dehydration-responsive element), T/G box, EE (evening element) [53,54] and ZFHDRS (ZFHD recognition sequence) [55], in their promoter regions (3000-bp upstream sequences from transcription start site [15]). Among these 15 motifs, 9 (ABRE2, CE3, DRE, ICEr2, MYBR, MYCR, G-box, T/G box and EE) were found in the promoter regions of the *GmNAC* candidate genes (Table S2). ICEr2 has been known as a cold-responsive *cis*-element [51], while ABRE2, CE3, DRE, MYBR, MYCR, G-box, T/G box and EE were identified as dehydration-inducible *cis*-motifs [51–54]. Consistent with their drought-inducible expression profile (Table 1), all six *GmNAC*s contain one or more dehydration-inducible *cis*-motifs in their promoter regions. Furthermore, our results indicated that in addition to dehydration-inducible *cis*-motifs, ICEr2 was also found in the promoter region of *GmNAC109*, suggesting that *GmNAC109* might also be induced by cold stress, and its promoter could be used in genetic engineering of soybean plants against not only drought but also cold stress.

## 3. Experimental Section

### 3.1. Plant Growth, Drought Treatment and Collection of Root Tissues

Two local soybean cultivars with contrasting drought-responsive phenotypes, DT51 (drought-tolerant) and MTD720 (drought-sensitive), were obtained from Legumes Research and Development Center and Can Tho University, Vietnam, respectively (Thu *et al.* unpublished data [56]). Plants were grown in plastic tubes (80 cm in height and 10 cm in diameter) filled with a mixture of soil, coconut fiber and cow pat (6:2:2 *w*/*w*) from Southern Fertilizer Company, under well-watered and greenhouse conditions (30/28 °C day/night temperatures, photoperiod of 12/12 h, and 60%–70% humidity) for 12 days. For drought treatment, the 12-day-old plants were non-irrigated for 15 days when the soil moisture content (SMC) was decreased to 5%–6%. For well-watered control, water was given to plants regularly once per day to maintain SMC at 65%–70%. After the drought treatment, control and drought-treated plants were removed carefully by cutting the plastic tubes longitudinally. The root tissues were collected, frozen in liquid nitrogen and stored at −80 °C until RNA isolation. The sampling process was performed with three biological replicates.

### 3.2. Total RNA Isolation and cDNA Synthesis

Total RNA was purified using Trizol reagent and PureLink RNA Mini Kit (Invitrogen, Carlsbad, CA, USA). DNaseI treatment was carried out using On-column PureLink DNase (Invitrogen, Carlsbad, CA, USA). RNA concentration was quantified twice for each sample using UV-vis spectrophotometer (Biotek, Winooski, VT, USA). First-stranded cDNA synthesis was performed using 1 μg of total RNA from each sample using cDNA Synthesis Kit (Thermo Scientific, Vilnius, Lithuania).

### 3.3. Real-Time Quantitative PCR

Gene-specific primer pairs for 23 *GmNAC* genes used in this study were showed in Table S1 with reference to [43]. For RT-qPCR of *GmNAC* genes, *Fbox* was used as reference gene [57]. RT-qPCR reactions were prepared in 25 μL final volume, which includes SYBR Green PCR Master Mix (Thermo Scientific, Vilnius, Lithuania), primer sets with final primer concentration of 0.4 μM/primer and 1 μL of cDNA template. The thermal profile of the RT-qPCR was 95 °C for 10 min, 40 cycles of 95 °C for 15 s and 60 °C for 1 min (Mastercycler^®^ ep *realplex*, Eppendorf, Hamburg, Germany). Dissociation curves were obtained using a thermal melting profile performed after the RT-qPCR cycle: 95 °C for 15 s followed by a constant increase in the temperature between 60 and 95 °C. The relative gene expression was calculated using the 2^−Δ^*^Ct^* method. In addition, fold change review between two conditions and cultivars was determined by the 2^−ΔΔ^*^Ct^* method. Background-corrected raw fluorescence data were exported from Mastercycler^®^ ep *realplex*, Eppendorf system (Hamburg, Germany) and analyzed in LinRegPCR software (version 2012.0, Academic Medical Center, Amsterdam, Netherlands, 2012) with a built-in baseline correction and amplification efficiency calculation [58]. The calculated amplification efficiencies of 24 specific primers (23 examined *GmNAC* and the *Fbox* reference gene) used in this study were showed in Table S1.

### 3.4. Discovery of *cis*-Regulatory Motifs in Promoter Regions of *GmNACs*

The identified positions of *cis*-motifs were described and presented in Table S2. The sequences of 16 well-known stress-inducible *cis*-motifs, including ABRE1, ABRE2, CE1, CRT, ICEr1, ICEr2, LTRE, MYBR, MYCR, NACR [8,51], G-box [52], CE3, DRE, T/G box, EE [53,54] and ZFHDR [55], were obtained from literature (Table S2). The 3000-bp promoter regions (3000-bp upstream sequences from transcription start site) of *GmNAC* genes were obtained from SoybeanTFDB [15]. The *cis*-motif search was performed as previously described [15].

### 3.5. Statistical Analysis of the Data

Drought-responsive genes were defined if the change in expression was at least 2-fold induction or repression under the water deficit treatment. When comparing expression of *GmNAC* genes between cultivars, differential expression ratio with at least 2-fold was considered as significant. The mean values of relative expression to *Fbox* were used to plot figures, and error bars on the top of bars represent standard errors of 3 biological replicates. The data were analyzed by Student’s *t*-test (one tail, unpaired, equal variance) to identify the statistical significance of differential gene expression within or between two cultivars under either normal or drought treatment with *p-*value < 0.05.

## 4. Conclusions

The results of this study suggested that in response to drought stress, transcriptional regulation of *GmNAC* genes may vary dependently on the genotypes of the cultivars. In addition, our study demonstrated that there is a positive correlation between the root-related expression of a subset *GmNAC* genes and the improved drought tolerance of DT51, in comparison with the drought-sensitive MTD720. This study also enabled us to identify a number of *GmNAC* candidates that may be subjected to in-depth functional characterizations aimed at improving drought tolerance in soybean.

## Figures and Tables

**Table 1.** Genes of Group A (gray) and Group B (white) with at least 2-fold up- or down-regulation by drought treatment in the roots of DT51 and/or MTD720. Data in italics indicate insignificant expression changes (≤2-fold and/or *p*-value ≥ 0.05) and classified as “unaltered” regulation. Data in bold indicate significant expression changes (≥2-fold and *p*-value < 0.05).

Nomenclature	Glyma ID	DT51	*p*-value	Regulation	MTD720	*p*-value	Regulation	Regulation in roots of W82 *
***GmNAC006***	*Glyma02g07700.1*	*1.4*	*0.1884*	Unaltered	**3.3**	**0.0085**	Down	Unaltered
***GmNAC011***	*Glyma02g26480.1*	**2.9**	**0.0074**	Up	**13.3**	**0.0098**	Down	Unaltered
***GmNAC019***	*Glyma04g38990.1*	*1.9*	*0.0299*	Unaltered	**9.1**	**0.0089**	Down	Up
***GmNAC038***	*Glyma06g15990.1*	*1.3*	*0.2121*	Unaltered	**2.2**	**0.0121**	Up	Up
***GmNAC043***	*Glyma06g38410.1*	**3.4**	**0.0478**	Up	**5.8**	**0.0001**	Up	Up
***GmNAC062***	*Glyma08g19300.1*	*1.9*	*0.2011*	Unaltered	**2.6**	**0.0340**	Up	Up
***GmNAC085***	*Glyma12g22880.1*	**5.5**	**0.0402**	Up	**5.9**	**0.0008**	Up	Up
***GmNAC092***	*Glyma12g35000.1*	**3.5**	**0.0126**	Up	*1.3*	*0.3609*	Unaltered	Up
***GmNAC095***	*Glyma13g05540.1*	**6.0**	**0.0289**	Up	**4.7**	**0.0447**	Up	Unaltered
***GmNAC099***	*Glyma13g31660.1*	**7.8**	**0.0097**	Up	**5.9**	**0.0114**	Up	Unaltered
***GmNAC101***	*Glyma13g35550.1*	**3.0**	**0.0026**	Up	**5.7**	**0.0009**	Up	Up
***GmNAC102***	*Glyma13g35560.1*	**2.1**	**0.0143**	Up	*1.0*	*0.4799*	Unaltered	Up
***GmNAC109***	*Glyma14g24220.1*	**3.3**	**0.0016**	Up	**2.3**	**0.0315**	Up	Up
***GmNAC148***	*Glyma20g04400.1*	**7.6**	**0.0027**	Down	**12.0**	**0.0151**	Down	Up
***GmNAC022***	*Glyma04g42800.1*	**10.5**	**0.0203**	Up	**2.1**	**0.0450**	Up	Unaltered
***GmNAC027***	*Glyma05g24910.1*	**3.2**	**0.0434**	Up	**7.9**	**0.0013**	Down	Unaltered
***GmNAC071***	*Glyma10g04350.1*	*1.6*	*0.1411*	Unaltered	**2.7**	**0.0207**	Down	Unaltered
***GmNAC083***	*Glyma12g13710.1*	*1.3*	*0.3577*	Unaltered	**2.0**	**0.0093**	Down	Unaltered
***GmNAC113***	*Glyma15g07620.1*	*1.6*	*0.1812*	Unaltered	**5.2**	**0.0016**	Down	Unaltered

* The information was obtained based on expression data of untreated and dehydrated root samples of W82 cultivar reported in Le *et al.* (2011) [43].

**Table 2.** Genes of Group A (gray) and Group B (white) with at least 2-fold differential expression ratio in DT51 *versus* MTD720 comparisons under unstressed and stressed conditions. The comparisons were performed individually for roots of the two cultivars under either normal or drought condition. Lower expression levels in DT51 compared to MTD720 were indicated by negative fold changes. Data in italics indicate insignificant expression changes (≤2-fold and/or *p*-value ≥ 0.05). Data in bold indicate significant expression changes (≥2-fold and *p*-value < 0.05).

Nomenclature	Glyma ID	Roots				Regulation	
	
		Normal	*p*-value	Drought	*p*-value	DT51	MTD720
***GmNAC006***	*Glyma02g07700.1*	−*1.5*	*0.1098*	**3.0**	**0.0386**	Unaltered	Down
***GmNAC011***	*Glyma02g26480.1*	*2.0*	*0.0707*	**76.6**	**0.0008**	Up	Down
***GmNAC019***	*Glyma04g38990.1*	*1.3*	*0.2279*	**23.3**	**0.0016**	Unaltered	Down
***GmNAC085***	*Glyma12g22880.1*	**2.3**	**0.0119**	**2.1**	**0.0467**	Up	Up
***GmNAC092***	*Glyma12g35000.1*	**2.4**	**0.0349**	**11.3**	**0.0045**	Up	Unaltered
***GmNAC095***	*Glyma13g05540.1*	**3.3**	**0.0129**	**4.2**	**0.0438**	Up	Up
***GmNAC101***	*Glyma13g35550.1*	**3.8**	**0.0002**	**2.0**	**0.0101**	Up	Up
***GmNAC109***	*Glyma14g24220.1*	**4.8**	**0.0075**	**6.9**	**0.0001**	Up	Up
***GmNAC148***	*Glyma20g04400.1*	**2.6**	**0.0039**	*4.2*	*0.1324*	Down	Down
***GmNAC017***	*Glyma04g33270.1*	**5.4**	**0.0443**	**9.8**	**0.0085**	Unaltered	Unaltered
***GmNAC022***	*Glyma04g42800.1*	*2.9*	*0.1947*	**14.3**	**0.0040**	Up	Up
***GmNAC027***	*Glyma05g24910.1*	−*1.3*	*0.1492*	**20.2**	**0.0194**	Up	Down
***GmNAC071***	*Glyma10g04350.1*	*2.0*	*0.0952*	**9.1**	**0.0061**	Unaltered	Down
***GmNAC083***	*Glyma12g13710.1*	*1.6*	*0.1714*	**4.4**	**0.0161**	Unaltered	Down
